# Concealed Around-the-Ear EEG Captures Cognitive Processing in a Visual Simon Task

**DOI:** 10.3389/fnhum.2017.00290

**Published:** 2017-06-08

**Authors:** Marlene Pacharra, Stefan Debener, Edmund Wascher

**Affiliations:** ^1^Leibniz Research Centre for Working Environment and Human Factors, TU Dortmund UniversityDortmund, Germany; ^2^Neuropsychology Lab, Department of Psychology, European Medical School, University of OldenburgOldenburg, Germany; ^3^Cluster of Excellence Hearing4All, University of OldenburgOldenburg, Germany

**Keywords:** around-the-ear EEG, cEEGrid, mobile EEG, Simon effect, stimulus-response correspondence

## Abstract

In theory, miniaturized systems such as the around-the-ear electrode arrays (cEEGrids) enable mobile monitoring of the electroencephalogram (EEG) in a variety of real life situations without interfering with the natural setting. However, the research benefit of such cEEGrid recordings critically depends on their validity. To investigate whether visual and motor processing are reflected in the cEEGrid-EEG, a direct comparison of EEG that was concurrently recorded with the cEEGrids and with a high-density cap setup was conducted. Thirteen participants performed a classic Simon task in which letters were presented laterally and a lateralized choice response was executed. N1, P1 and P300 event-related potential (ERP) waveforms were extracted from cEEGrid-EEG: they were found to be strongly correlated with corresponding waveforms extracted from cap-EEG but with lower signal strength and lower signal-to-noise-ratio (SNR). Event-related lateralizations (ERLs) recorded at posterior scalp sites were well reflected in middle cEEGrid pairs. Moreover, the effect size of the Simon correspondence effect on the extracted ERLs was similar between the two systems. However, lateralizations at central cap sites were less well reflected in the cEEGrid-EEG indicating a difficulty in capturing motor response preparation and execution. These results show that well-described visual and cognitive ERPs and ERLs can be measured using the cEEGrids, while motor-related cortical potentials are not well captured. This study further demonstrates the potential and possible limitations of unobtrusive cEEGrid-EEG recordings.

## Introduction

Due to their size and limited portability, most brain imaging technologies are restricted to highly artificial and controlled laboratory conditions. Consequences are a limited range of recording settings (excluding e.g., most everyday life contexts and activities), constricted natural behavior by participants, and possibly low ecological validity (compare Gramann et al., 2011; Ladouce et al., 2017). In the last 10 years, interest in investigating the electroencephalogram (EEG) in natural walking situations (Debener et al., 2012) or simulated workplaces (Wascher et al., 2014, 2015) has increased which is facilitated by technological advances in the mobile recording of the EEG. However, bulky and conspicuous cap-EEG systems have low social acceptability (e.g., due to increased experimental awareness by the wearer and others) and low wearer comfort which so far interfere with recording EEG in truly natural situations.

This has triggered the recent development of intra-auricular and miniaturized around-the-ear EEG systems: on the one hand, personalized and generic earpieces can be used to place electrodes in the outer ear canal providing good signal quality with low signal-to-noise-ratio (SNR) in a range of settings (Kidmose et al., 2013; Mikkelsen et al., 2015; Goverdovsky et al., 2016). On the other hand, the newly available cEEGrid is a flex-printed c-shaped multi-electrode array that neatly fit behind the ear of a wearer and is thus hardly visible (Debener et al., 2015). Debener et al. (2015) showed that the cEEGrid enables the acquisition of EEG signals (“cEEGrid-EEG”) over many hours without significant discomfort or distraction for the wearer. Moreover, two further studies with a focus on auditory processing demonstrated the validity of cEEGrid-EEG for capturing auditory selective attention effects (Bleichner et al., 2016; Mirkovic et al., 2016). At present, it is not well known whether signals from other sensory modalities or motor processes are captured as well in cEEGrid-EEG. If this could be demonstrated, other applications for the cEEGrid-EEG — beyond auditory rehabilitation — would arise. Brain-computer-interfaces (BCI) as well as applications in neuroergonomics would benefit from the possibility of covertly and continuously monitoring neuronal activity (Mehta and Parasuraman, 2013).

In order to fill this gap, we aimed to provide evidence that cEEGrid and traditional cap-EEG recordings provide comparable event-related potentials (ERPs) and event-related lateralizations (ERLs, Wascher and Wauschkuhn, 1996) in response to visual stimulation. Theoretically, the limited spatial sampling of the cEEGrids (e.g., short distance between cEEGrid electrodes and reference) should result in smaller amplitudes and impair the ability to capture distant electrical signals, due to the subtraction of increased common (far-field) mode (compare Debener et al., 2015). However, it has been assumed that large-distance cap-EEG channels and small-distance cEEGrid channels have similar SNR due to the opposing effects of electrode distance on common mode and common far-field noise (Bleichner et al., 2015; Debener et al., 2015).

Thus, the amount of spatial information and the SNR that is present in the cEEGrid recordings compared to cap-EEG needs to be experimentally quantified, preferably in studies comparing cEEGrid and cap-EEG effects in concurrent recordings. The aim of the present study was to investigate the similarity of ERPs and ERLs derived from cap-EEG and cEEGrid-EEG. A spatial stimulus-response correspondence (Simon) task was used for this purpose, as it offers the opportunity to examine the neural correlates of early and later stages of visual processing as well as lateralized motor response preparation (Wascher et al., 2001; Leuthold, 2011). The change in spatial correspondence in this task requires considerable response control from the participants (Möckel et al., 2015). Spatial correspondence between target and response location can accelerate responses, while spatial non-correspondence can decelerate responses (Simon, 1990).

## Materials and Methods

### Participants

Fourteen right-handed participants at the age of 18–30 years were recruited for this study. One participant was excluded from data analysis, as cap-EEG pre-processing resulted in the elimination of all trials of one experimental condition in this participant. Thus, the study sample consisted of 13 participants (4 male, mean age = 24.4 years). All participants reported to be free of past or present neurological or psychiatric conditions and had normal or corrected-to-normal vision.

Prior to the experiment, all participants provided written informed consent in accordance with the Declaration of Helsinki. The study and experimental protocol were approved by the local ethics committee of the Leibniz Research Centre for Working Environment and Human Factors and conducted in accordance with the Declaration of Helsinki. Participants received course credit or a financial compensation for their participation (approximately 30 €).

### Stimuli and Task

In this version of the Simon task (compare Simon, 1990), one of two letters (“H” or “N”) was presented randomized either on the left or the right side of a fixation cross for 200 ms (both sides equally frequent). The visual angle from the center of the letters to the fixation cross was 2°. Twenty percent of the 600 task trials were NoGo trials indicated by a diamond around the letter (1.41° visual angle) and 80% of trials were Go trials indicated by a circle around the letter (1.1° visual angle).

Only Go trials could be either corresponding or non-corresponding: in Go trials, participants had to press the right button of a force measuring device with their right thumb when the letter “H” appeared. They had to press the left button with their left thumb when the letter “N” appeared. Participants had to ignore the location where the letter was presented. Thus, a Go trial was corresponding when the side of stimulus presentation and response execution was the same. A Go trial was non-corresponding when the side of stimulus presentation and response execution was different. The inter-stimulus interval was 1720–2020 ms (jittered). After half of the trials, participants had a short break.

### EEG Data Recording

EEG was recorded simultaneously with two systems: on the one hand, EEG was recorded with two cEEGrids (TMSi, Oldenzaal, Netherlands) which were positioned around the participant’s left and right ear respectively (“cEEGrid-EEG”). On the other hand, EEG was recorded with a traditional 64-channel EEG cap (“cap-EEG”).

**Figure 1:**
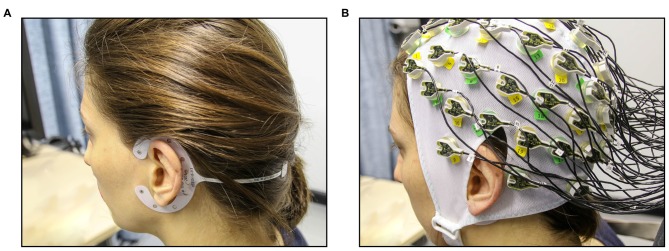
Experimental setup for the concurrent cEEGrid-EEG and cap-EEG recordings. **(A)** First, cEEGrids were fitted around the left and the right ear of the participant. **(B)** Next, the 64-channel EEG cap was fitted (cEEGrids remain underneath cap).

For the cEEGrid-EEG, the skin around the ear was cleaned with abrasive gel and alcohol (for cEEGrid preparation, see http://ceegrid.com). A small amount of electrolyte gel (SuperVisc, Easycap GMBH, Munich, Germany) was applied to the cEEGrid electrodes. With the help of a double-sided adhesive, the cEEGrid was placed around the ear without touching the auricle (see Figure 1A). This procedure is comparable with previous studies investigating the validity of cEEGrid recordings (Debener et al., 2015; Bleichner et al., 2016; Mirkovic et al., 2016). As cEEGrid’s placement angle is adjusted to fit a participant’s ear anatomy (see Bleichner and Debener, 2017), it can be assumed that cEEGrid positioning varied slightly between participants as in previous studies (Mirkovic et al., 2016).

There was a hardwired connection between the two cEEGrids and a QuickAmp DC-amplifier (Brain Products GmbH, Gilching, Germany) set to low pass filtering of 280 Hz. Sampling rate was 1 kHz and resolution was 24 bits. As in a previous study (Debener et al., 2015), the two electrodes in the middle of the right cEEGrid served as ground and online reference respectively (R4a, R4b). Offline cEEGrid-EEG data were re-referenced to algebraically linked mastoids (R4b, L4b). The cEEGrid electrode on the left side that had no homologous counterpart (e.g., ground electrode of the right cEEGrid) was discarded from the data analysis: this yielded a symmetrical 16 channel montage (8 channels per ear: L1–L8 and R1–R8).

For the acquisition of the cap-EEG, a standard 64-channel cap was used (Easycap GMBH, Munich, Germany) with Ag/AgCl electrodes that were attached according to the extended 10/20 system (Pivik et al., 1993). The cap was connected to a BrainAmp DC-amplifier (Brain Products, Gilching, Germany) and a low pass filter of 250 Hz was applied. Sampling rate was 1 kHz with resolution of 16 bits. The two cap electrodes on the mastoid bones (TP 10 and TP 9) could not be used due to overlap with the cEEGrids (compare Figure 1B) and were thus not available as local referencing sites. Therefore, cap-EEG data were recorded against a common average reference as by Mirkovic et al. (2016).

### EEG Data Analysis

EEG data was analyzed using EEGLAB version v13.6.5b and custom built scripts in Matlab 2016b (The Mathworks Inc., Natick, MA, USA).

cEEGrid-EEG and cap-EEG were filtered offline with a high-pass filter of 1 Hz and a low-pass filter of 25 Hz and resampled at 250 Hz. For stimulus-locked analysis, cEEGrid-EEG and cap-EEG data were segmented in intervals of −500 ms to 1000 ms after stimulus presentation. For response-locked analysis, data were segmented in corresponding intervals (−500 ms to 1000 ms) after response execution. For both stimulus-locked and response-locked analysis a 200 ms interval before stimulus presentation was set as baseline.

After performing statistics-based artifact removal as implemented in EEGLAB (standard deviation: 4), an independent component analysis (ICA) was applied separately to cEEGrid-EEG and cap-EEG. Matrices of independent components (ICs) were projected back to cEEGrid- and cap-EEG data filtered with a high-pass filter of 0.1 Hz and a low-pass filter of 25 Hz (resampled at 250 Hz). Data were epoched as previously described. Statistics-based artifact removal (standard deviation: 4) resulted in the rejection of 26.3% of trials in cEEGrid-EEG data and 28.8% trials in cap-EEG data, *t*_(25)_ = −1.3, *p* = 0.222.

For cap-EEG, the EEGLAB plugin ADJUST was used to identify and reject ICs reflecting artifacts (Mognon et al., 2011). Using a standardized procedure (see Debener et al., 2005; Wascher et al., 2015), the remaining ICs were tested for biological plausibility: component plausibility was quantified by the goodness of fit for modeling the individual IC scalp map with a single equivalent current dipole. For this, individual component maps were subjected to an automatic source localization algorithm implemented in EEGLAB (DIPFIT, see Oostenvelt et al., 2003) which refers to a standard four-shell spherical head model. Individual ICs, which had a residual variance of more than 50%, were automatically removed from the cap-EEG data (on average 42.0% of all ICs removed).

**Figure 2:**
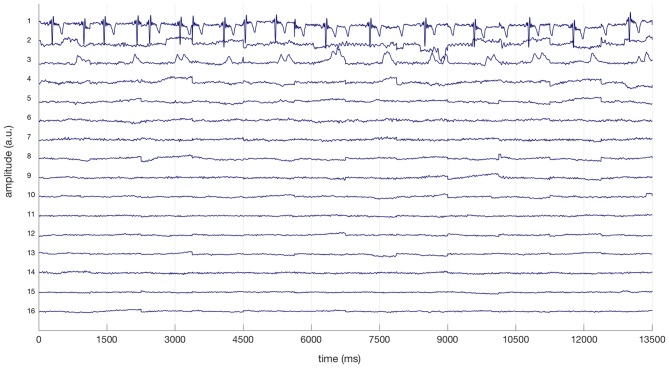
Excerpt of a linear decomposition of cEEGrid data with independent component analysis (ICA) for a representative participant. Based on the prominent QRS complexes, the first independent component (IC) represents heart-electrical activity. The second and third ICs reflect horizontal eye movements and regular eye blinks respectively (compare Bleichner and Debener, 2017). ICs 1, 2 and 3 were removed from cEEGrid data.

The electrocardiogram (EKG) is often reflected in specific ICs extracted from cEEGrid-EEG and eye-artifact processing is strongly advised with all cEEGrid data (see Bleichner and Debener, 2017). Therefore, ICs reflecting EKG based on prominent QRS complexes and regular eye artifacts were removed from cEEGrid-EEG data after visual inspection (see Bleichner and Debener, 2017). All EEG data was also specifically checked for ICs reflecting horizontal eye movements and relevant ICs were removed. Figure 2 illustrates this procedure for the cEEGrid data for one representative participant: three ICs (1, 2, 3) were removed from the cEEGrid data reflecting heart-electrical activity, horizontal eye movements and eye blinks respectively. For cEEGrid data, this procedure resulted in the exclusion of on average 14.7% of all components from individual data sets. The ratio of remaining ICs after exclusion was therefore significantly greater in the cEEGrid-EEG (85.3%) compared to the cap-EEG after the automatic procedure (58.0%), *t*_(25)_ = −12.8, *p* < 0.001.

Stimulus-locked ERPs and stimulus- and response-locked ERLs were analyzed. ERLs were computed using a double-subtraction method (Eimer, 1998): in detail, first difference amplitudes were computed between ERPs recorded over the left and the right hemisphere (e.g., ERP [C1] − ERP [C2]) separately for left and right events. The resulting difference waveform for right events was then subtracted from the difference waveform for left events:

$${\text{ERL = [ERP(left hemisphere) − ERP(right hemisphere)]}}_{\text{left event}}{\text{− [ERP(left hemisphere) − ERP(right hemisphere)]}}_{\text{right event}}$$

Stimulus-locked ERLs were calculated for the symmetrical electrode pairs PO7/PO8, C1/C2, T7/T8 of the cap-EEG. The ERL over C1/C2 is comparable to the classical lateralized readiness potential (LRP) recorded over C3′/C4′ (see Eimer, 1998; Wascher et al., 2001). While the ERL over T7/T8 is not typically of interest in the Simon task (but Wascher et al., 2001), it was included because of the close proximity between the cap electrodes T7/T8 and the cEEGrids. Due to the interest in motor responses, response-locked ERLs were additionally computed for the cap electrode pair C1/C2 and T7/T8. For the cEEGrid data, stimulus- and response-locked ERLs were calculated using the same double-subtraction method for all symmetrical (e.g., L1/R1) cEEGrid electrode pairs.

Due to smaller amplitudes of ERP waveforms for cEEGrid-EEG compared to cap-EEG data (Debener et al., 2015), waveshape similarity between systems is often difficult to evaluate by visual inspection. Therefore, *z*-standardized ERP waveforms are also presented which were calculated by subtracting the mean activation over the total time interval from the individual activation at each time point and then dividing the difference by the standard deviation over the time interval.

### Statistical Analysis

#### Correlational Analysis between cEEGrid-EEG and cap-EEG

In a first step, the similarity in ERP and ERL waveform morphology between cEEGrid-EEG and cap-EEG recordings was determined. For early visual processing (e.g., N1, P1), a Pearson’s correlation coefficient was calculated between the mean activity at each cEEGrid electrode and the averaged activity at PO7 and PO8 over all time points 50 ms to 250 ms after stimulus presentation for each individual subject. To test waveshape similarity in the later processing stages (e.g., P300), a Pearson’s correlation coefficient was calculated for each subject between the mean activity at each cEEGrid electrode and the mean activity at Pz over all time points 250 ms to 550 ms after stimulus presentation. For stimulus-locked ERLs, mean activity in the time interval between 150 ms and 600 ms was analyzed separately for corresponding and non-corresponding trials. Likewise, for response-locked ERLs, mean activity in the time interval between −200 and 200 ms after response execution was analyzed separately for corresponding and non-corresponding trials.

For a group level estimate of similarity, subject-wise correlation coefficients for the different time windows were first Fisher *z*-transformed and then averaged (Corey et al., 1998). Using the method suggested by Bonett (2008), 95%-confidence intervals (95%-CI) were constructed around average correlation coefficients. Average correlation coefficients and 95%-CI limits were subjected to the inverse Fisher’s *z-transformation* to increase ease of interpretability of the results.

#### Comparison of Component Features and SNR between cEEGrid-EEG and cap-EEG

An EEGLAB plugin (ERPlab, see Lopez-Calderon and Luck, 2014) was used to extract peak amplitude and 50% fractional area latency for P1 (50–125 ms) and N1 (100–250 ms) at averaged cap channels PO7/PO8 and T7/T8. Peak amplitude and 50% fractional area latency for P300 (250–550 ms) at cap Pz and at averaged cap channels T7/T8 were determined using the same tool. Based on previous studies (e.g., Wascher and Wauschkuhn, 1996; Wascher et al., 2001), it was expected that stimulus-locked posterior and central ERL waveforms should have an earlier peak (150–300 ms) and a later peak (posterior: 250–400 ms; central: 250–600 ms), while temporal ERLs should have one peak (150–300 ms). Therefore, peak amplitude and 50% fractional area latency were extracted from stimulus-locked ERL waveforms for these time intervals, while parametrization for response-locked ERLs was restricted to one time interval (−200 to 200 ms after the response).

Corresponding parametrization of P1, N1 and P300 and ERLs was also performed for data collected at cEEGrid electrodes. Differences in peak amplitude and 50% fractional area latency between cEEGrid-EEG and cap-EEG were evaluated with paired *t* tests.

The SNR was calculated by dividing the mean ERP amplitude (P1, N1, P300) by the standard deviation in the pre-stimulus interval (Debener et al., 2012). Differences in ERP SNR between cEEGrid-EEG and cap-EEG were evaluated with paired *t* tests.

#### Comparison of Effect Sizes between cEEGrid-EEG and cap-EEG

The stimulus-locked spatial correspondence (Simon) effect on ERLs recorded from cap- and cEEGrid-EEG was quantified using the Cohen’s *d* effect size measure. For every latency of the respective time windows, Cohen’s *d* was computed as the mean difference between the ERLs for the corresponding and non-corresponding condition at this time point divided by the respective pooled standard deviation. Based on previous studies (compare Wascher and Wauschkuhn, 1996; Wauschkuhn et al., 1998), response-locked central ERLs were not expected to be different in the corresponding and non-corresponding condition of the Simon task. Thus, in the response-locked analysis, a Cohen’s *d* effect size measure was computed that quantified the difference between no activation and the mean ERLs of both conditions instead of the Simon condition effect.

## Results

### ERP Analysis

#### Early Visual Processing

**Figure 3:**
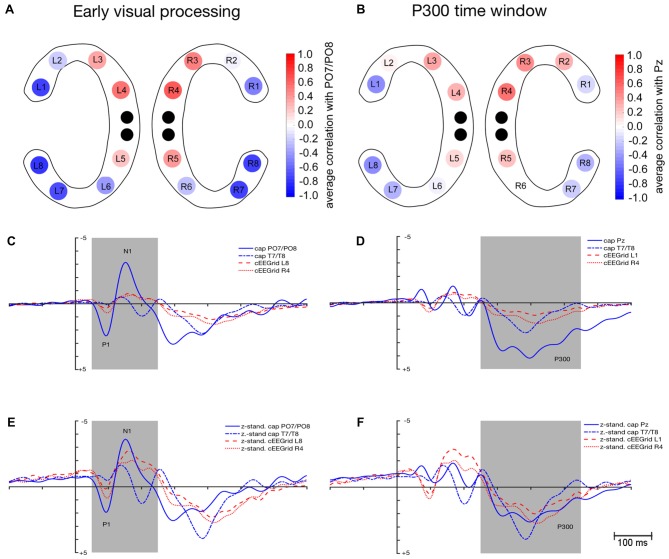
Similarity in event-related potential (ERP) waveform morphology between ERPs extracted from cEEGrid-EEG and cap-EEG for the early visual processing (50–250 ms) and P300 time window (250–550 ms). **(A)** Color-coded average correlations (*p* ≤ 0.05) between mean activity at each cEEGrid electrode and at cap PO7/PO8. **(B)** Color-coded average correlations (*p* ≤ 0.05) between mean activity at each cEEGrid electrode and at cap Pz. **(C)** Grand-average ERPs as recorded from cap PO7/PO8, cap T7/T8 (inverse), cEEGrid L8 (inverse) and cEEGrid R4. **(D)** Grand-average ERPs as recorded from cap Pz, cap T7/T8 (inverse), cEEGrid L1 (inverse) and cEEGrid R4. **(E)** *z-standardized* grand-average ERPs as recorded from cap PO7/PO8, cap T7/T8 (inverse), cEEGrid L8 (inverse) and cEEGrid R4. **(F)** *z*-standardized grand-average ERPs as recorded from cap Pz, cap T7/T8 (inverse), cEEGrid L1 (inverse) and cEEGrid R4. Black dots in **(A,B)** represent reference and ground electrodes of the cEEGrids. Shaded areas in **(C–F)** represent time windows for which similarity between ERPs extracted from the cap- and the cEEGrid-EEG was analyzed.

As depicted in Figure 3A, average correlation coefficients ($\bar{r}$) between ERPs recorded at cEEGrids and at cap PO7/PO8 were highest for the outer and middle cEEGrid electrodes in the early visual processing window. Numerically, average correlation coefficients ranged from −0.79 to 0.64 with negative correlations found with outer cEEGrid electrodes and positive correlations with middle cEEGrid electrodes. Here, the highest average correlations were observed between the activity at PO7/PO8 on the one hand and the left outer (L8, $\bar{r}$ = −0.79, 95%-CI: [−0.83, −0.73]) and the right middle cEEGrid electrode (R4, $\bar{r}$ = 0.64, 95%-CI: [0.57, 0.70]) on the other hand.

Figure 3C shows the grand-average waveforms that were recorded at cap PO7/PO8 and T7/T8 as well as at the cEEGrid electrodes L8 and R4. Figure 3E shows the *z*-standardized ERPs for the same electrodes. Differences in signal strength are apparent between cap PO7/PO8 and cEEGrids (see Figure 3C): peak amplitudes for P1 were significantly higher when recorded at PO7/PO8 compared to cEEGrid L8, *t*_(12)_ = 4.1, *p* = 0.001, and cEEGrid R4, *t*_(12)_ = 3.7, *p* = 0.003. Also, peak amplitudes for N1 were significantly higher when recorded at PO7/PO8 compared to cEEGrid L8, *t*_(12)_ = −5.8, *p* < 0.001, and cEEGrid R4, *t*_(12)_ = −4.2, *p* = 0.001. No differences in signal strength emerged between cEEGrid electrodes and cap electrodes close to the cEEGrids (i.e., T7/T8): differences in P1 and N1 amplitude between cap T7/T8 and cEEGrid L8 and R4 were not significant, paired *t* tests *p* > 0.05.

No significant differences in 50% fractional area latency emerged regarding P1 measured at PO7/PO8 and T7/T8 compared to cEEGrid L8 and cEEGrid R4, all paired *t* tests *p* > 0.05. However, 50% fractional area latencies of N1 were significantly longer when measured at cEEGrid L8 compared to PO7/PO8, *t*_(12)_ = −2.3, *p* = 0.034, but not significantly different from T7/T8, *t*_(12)_ = 0.7, *p* = 0.451. There was no significant difference in N1 latency when comparing cEEGrid R4 with cap PO7/PO8 and when comparing cEEGrid R4 with cap T7/T8, paired *t* tests *p* > 0.05.

The P1 SNR was significantly higher at cap PO7/PO8 compared to cEEGrid L8, *t*_(12)_ = −5.1, *p* < 0.001 and cEEGrid R4, *t*_(12)_ = −4.8, *p* < 0.001. Moreover, N1 SNR was significantly higher at cap PO7/PO8 compared to cEEGrid L8, *t*_(12)_ = −3.5, *p* = 0.004, and cEEGrid R4, *t*_(12)_ = −3.0, *p* = 0.011. Mean P1 SNR over all cEEGrid channels was 3.6 compared to 21.8 at PO7/PO8, while mean N1 SNR over all cEEGrid channels was 7.8 compared to 35.5 at PO7/PO8. P1 and N1 SNR at cap electrodes close to cEEGrids (i.e., T7/T8) were comparable to SNR at cEEGrids, paired *t* tests *p* > 0.05.

#### P300 Time Window

As depicted in Figure 3B, a similar spatial correlation pattern as for the early visual processing window emerged with respect to the P300 time window: average correlation coefficients between ERPs recorded at cEEGrids and at cap Pz were highest at the outer and middle cEEGrid electrodes with negative correlations for outer and positive correlations for middle cEEGrid electrodes. Numerically, these average correlation coefficients ranged from −0.46 to 0.54. Here, the highest average correlations were observed between the activity at Pz on the one hand and the left outer (L1, $\bar{r}$ = −0.46, 95%-CI: [−0.52, −0.40]) and the right middle cEEGrid electrode (R4, $\bar{r}$ = 0.54, 95%-CI: [0.48, 0.59]) on the other hand.

Figure 3D shows the grand-average P300 waveforms that were recorded at cap Pz and T7/T8 as well as at the cEEGrid electrodes L1 and R4. Figure 3F shows the *z*-standardized ERPs for the same electrodes. In Figure 3D, differences in signal strength with significantly higher P300 peak amplitudes at Pz compared to cEEGrid L1, *t*_(12)_ = 5.7, *p* < 0.001, and cEEGrid R4, *t*_(12)_ = 4.1, *p* = 0.001, are apparent.

P300 amplitudes recorded at cap electrodes near cEEGrids (T7/T8) were not significantly different from P300 amplitudes recorded at cEEGrid L1 and R4, paired *t* test *p* > 0.05. No significant differences in 50% fractional area latency emerged between P300 measured at cap Pz and T7/T8 compared to P300 measured at cEEGrid L1 and cEEGrid R4, paired *t* tests *p* > 0.05.

The P300 SNR was significantly higher at cap Pz (*M* = 18.2) compared to cEEGrid L1 (*M* = 11.6), *t*_(12)_ = −3.4, *p* = 0.005, but not significantly different from cEEGrid R4 (*M* = 13.8), *t*_(12)_ = −1.2, *p* = 0.237. Mean P300 SNR over all cEEGrid channels was 9.5 and not significantly different from P300 SNR at cap electrodes close to cEEGrids (T7/T8), paired *t* test *p* > 0.05.

### ERL Analysis

**Figure 4:**
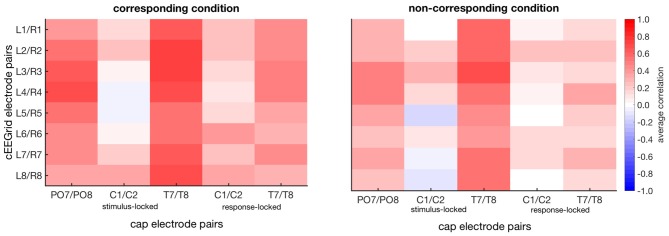
Color-coded average correlations between stimulus-locked event-related lateralizations (ERLs) recorded from symmetrical cEEGrid pairs (left/right cEEGrid) and ERLs recorded from cap pairs 150 ms to 600 ms after stimulus presentation in the corresponding and non-corresponding condition (first three columns of each plot). Also, color-coded average correlations between response-locked ERLs recorded from symmetrical cEEGrid pairs (left/right cEEGrid) and ERLs recorded from the cap C1/C2 and T7/T8 pair −200 ms to 200 ms after response execution (last two columns of each plot). Only significant correlations are shown (*p* ≤ 0.05).

Figure 4 shows the average correlations between the ERLs recorded at the symmetrical cEEGrid channels and the ERLs recorded at the cap electrode pairs PO7/PO8, C1/C2 and T7/T8.

#### Posterior ERLs

For posterior ERLs recorded at the PO7/PO8 cap pair, the highest correlations were found with the ERLs recorded over middle cEEGrid pairs (see Figure 4), in particular the L4/R4 pair (corresponding $\bar{r}$ = 0.65, 95%-CI: [0.61, 0.69]; non-corresponding $\bar{r}$ = 0.48, 95%-CI: [0.44, 0.52]).

**Figure 5:**
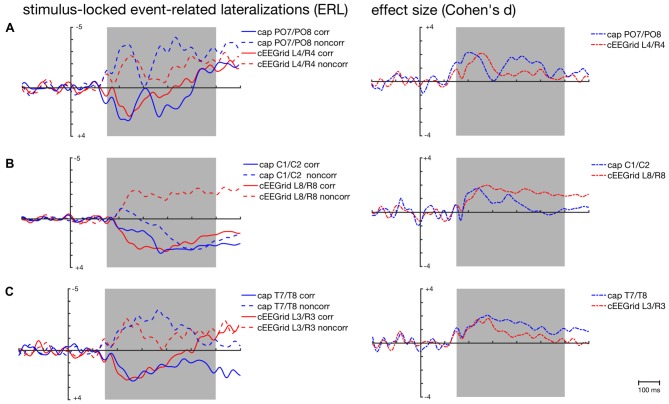
Stimulus-locked grand-average ERLs for the corresponding (corr) and non-corresponding (noncorr) condition and associated effect sizes (Cohen’s *d*) for the Simon condition effect (corr—noncorr) over time as recorded over posterior **(A)**, central **(B)** and temporal cap sites **(C)** and cEEGrid electrodes. Shaded areas depict time windows for which similarity between ERLs extracted from the cap- and cEEGrid-EEG was analyzed.

The respective grand-average ERL waveforms for the PO7/PO8 and the L4/R4 pair are depicted in Figure 5A. As expected, corresponding and non-corresponding ERL waveforms exhibit two prominent peaks. Peak amplitudes and 50% fractional area latencies for both time windows (150–300 ms; 250–400 ms) were not significantly different between the ERLs extracted from cap-EEG and cEEGrid-EEG in both conditions, paired *t* tests *p* > 0.05.

The size of the spatial correspondence (Simon) effect over time on ERLs recorded over PO7/PO8 and L4/R4 was similar (see Figure 5A).

#### Central ERLs

As depicted in Figure 4, average correlations between the ERLs recorded over central cap sites and the ERLs recorded over the symmetrical cEEGrid pairs were low in the stimulus- and response-locked analysis. In the stimulus-locked analysis, the highest correlations were found between the ERLs recorded over C1/C2 and the ERLs recorded over the symmetrical L8/R8 pair (corresponding $\bar{r}$ = 0.34, 95%-CI: [0.29, 0.38]; non-corresponding $\bar{r}$ = −0.08, 95%-CI: [−0.13, −0.02]).

Figure 5B shows the weak correspondence between the ERLs extracted from the electrode cap pair C1/C2 and the cEEGrid pair L8/R8. Fifty percent fractional area latencies in the two conditions and in the two time windows did not differ significantly between ERLs extracted from cap-EEG and cEEGrid-EEG. But significant differences in peak amplitude emerged for the corresponding condition in the early time window, *t*_(12)_ = −2.7, *p* = 0.020, and for the non-corresponding condition in the early, *t*_(12)_ = 3.7, *p* = 0.003, and late time window, *t*_(12)_ = 3.7, *p* = 0.003.

This is also reflected in the time course of the respective spatial correspondence (Simon) effect sizes (see Figure 5B). While there are two maxima in the time course of the cap-derived effect sizes corresponding to the major inflections in the ERLs for the corresponding and non-corresponding condition respectively, there is only one maximum in the course of the cEEGrid-EEG derived effect sizes.

**Figure 6:**
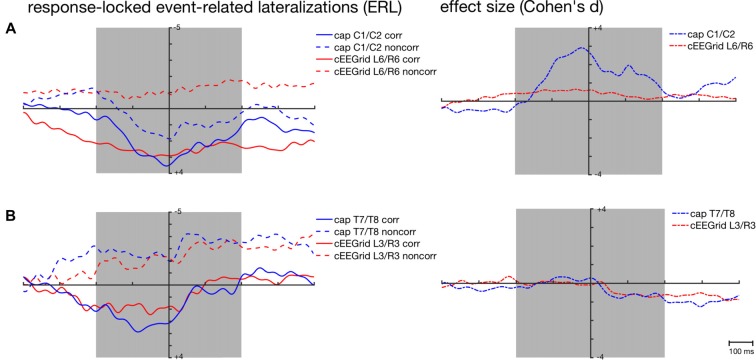
Response-locked grand-average ERLs for the corresponding (corr) and non-corresponding (noncorr) condition and associated effect sizes (Cohen’s *d*) for the response-locked effect ((corr + noncorr) − 0) over time as recorded over central **(A)** and temporal cap sites **(B)** and cEEGrid electrodes. Shaded areas depict time windows for which similarity between ERLs extracted from the cap- and cEEGrid-EEG was analyzed.

For the response-locked ERLs recorded over the C1/C2 electrode cap pair, the highest correlations were found with the ERLs recorded over the symmetrical L6/R6 pair (corresponding $\bar{r}$ = 0.38, 95%-CI: [0.33, 0.43]; non-corresponding $\bar{r}$ = 0.16, 95%-CI: [0.10, 0.20]). Figure 6A confirms the weak correspondence between response-locked ERLs extracted from cap-EEG and cEEGrid-EEG for both conditions.

This is also reflected in the effect sizes (Figure 6A) with stronger response-locked effect sizes for the cap-derived ERLs (C1/C2) than for the cEEGrid-EEG derived ERLs (L6/R6). However, both peak amplitudes and 50% fractional area latencies in both conditions did not differ significantly between response-locked ERLs extracted from cap-EEG and cEEGrid-EEG, all paired *t* tests *p* > 0.05.

#### Temporal ERLs

As depicted in Figure 4, average correlations between the ERLs recorded over temporal cap sites close to the cEEGrids and the ERLs recorded over the symmetrical cEEGrid pairs were high in the stimulus-locked and response-locked analysis. In the stimulus-locked analysis, the highest correlations were found between the ERLs recorded over T7/T8 and the ERLs recorded over the symmetrical L3/R3 pair (corresponding $\bar{r}$ = 0.73, 95%-CI: [0.69, 0.76]; non-corresponding $\bar{r}$ = 0.66, 95%-CI: [0.62, 0.70]). In the response-locked analysis, the highest correlations were also found between the ERLs recorded over T7/T8 and the ERLs recorded over the symmetrical L3/R3 pair (corresponding $\bar{r}$ = 0.51, 95%-CI: [0.46, 0.56]; non-corresponding $\bar{r}$ = 0.13, 95%-CI: [0.07, 0.18]).

Figures 5C, 6B show the strong correspondence between ERLs extracted from the electrode cap pair T7/T8 and the cEEGrid pair L3/R3. In the stimulus-locked analysis, ERL extracted from T7/T8 had longer 50% area latencies in the corresponding, *t*_(12)_ = 3.6, *p* = 0.004, and non-corresponding condition, *t*_(12)_ = 3.5, *p* = 0.005, than ERLs extracted from cEEGrid L3/R3. 50% fractional area latencies in the response-locked analysis and peak amplitudes in both types of analyses did not differ significantly between ERLs extracted from the T7/T8 cap pair and ERLs extracted from the L3/R3 pair, all paired *t* tests *p* > 0.05. The size of the spatial correspondence (Simon) effect over time on ERLs recorded over T7/T8 and R3/L3 was similar (see Figure 5A), while the response-locked effect over time on both ERLs recorded over T7/T8 and L3/R3 was close to zero (see Figure 6B).

## Discussion

In this study, the ERP and ERL waveforms extracted from cap-EEG generally showed the specific time and condition dynamics that were expected for a horizontal Simon task (Valle-Inclán, 1996; Wascher and Wauschkuhn, 1996; Wascher et al., 2001; Leuthold, 2011). In detail, P300 peak latencies were in the range of 350 ms as observed previously (see Leuthold, 2011), although P300 amplitudes were around 2–3 μV smaller than expected from previous studies (Wascher and Wauschkuhn, 1996; Leuthold, 2011). ERLs over occipital sites indicated a short-lived positive lateralization in corresponding trials and a negative lateralization in non-corresponding trials between 150 ms and 250 ms, which temporally coincided with an initial smaller ERL peak over C1/C2 (compare Eimer, 1998; Leuthold, 2011). Considering that ERLs calculated using the double subtraction method are always twice as large and of the opposite polarity than ERLs calculated using the averaging method (Eimer, 1998), peak amplitudes for cap ERLs were 1–2 μV smaller than expected based on the data by Wascher and Wauschkuhn (1996).

Sensory and cognitive-driven ERP and ERL waveforms recorded with the cEEGrid-EEG generally showed similar shape and time characteristics as the waveforms recorded with the cap-EEG. Considering the differences in placement of references and electrodes between the cEEGrid-EEG and the traditional cap-EEG system, the correspondence of ERP waveforms with correlations up to −0.79 (95%-CI: [−0.83, −0.73]) over specific time windows seems remarkable. Reference-free posterior and temporal ERL waveforms (Wascher and Wauschkuhn, 1996) also exhibited strong correlations across the two systems. Moreover, the size of the spatial correspondence effect over time on posterior ERLs derived from cap-EEG and on ERLs derived from cEEGrid-EEG was comparable.

At first glance, these results are in line with previous experimental results concerning the good validity of sensory-driven signals in the cEEGrid-EEG (Bleichner et al., 2016; Mirkovic et al., 2016). However, in contrast to our expectations, we could not show that large-distance cap-EEG channels and small-distance cEEGrid channels have similar SNR for well-known ERPs (compare Bleichner et al., 2015; Debener et al., 2015). The observed lower SNR and lower signal strength in the cEEGrid recordings compared to established occipital and central cap-EEG positions may be of concern for the practical application of the cEEGrids in mobile settings (with additional motion-related noise, see Gramann et al., 2011; Ladouce et al., 2017) or BCI.

On the one hand, Kidmose et al. (2013) showed that SNR in cap and intra-auricular EEG are comparable highlighting a possible advantage of in-ear compared to around-the-ear recordings. On the other hand, a recent study by Wang et al. (2016) also observed lower SNR from electrodes behind the ear compared to electrodes on occipital areas for steady-state visual evoked potentials. But the authors could show that SNR was sufficient for an online BCI application. In our study, differences in reference placement between cap- and cEEGrid-EEG systems and a lack of more sophisticated artifact pre-processing algorithms for cEEGrid data (compare Bleichner and Debener, 2017) may have contributed to low signal strength and SNR.

The current study shows that well-known lateralized motor preparation and motor control processes were not adequately captured by the cEEGrid-EEG. Symmetrical cEEGrid pairs were unable to capture the typical dynamics of the classical LRP in the Simon task that is usually observed over cap pairs C3′/C4′ or C1/C2 (Eimer, 1998; Leuthold, 2011). This resulted in a negligible size of the response-locked effect in the cEEGrid-EEG compared to the traditional cap-EEG recorded over central scalp sites. The well-investigated LRP in the Simon task has been linked to a covert response preparation and is mainly generated in the primary motor cortex (M1, Leuthold and Jentzsch, 2002). Thus, the cEEGrids may either be unable to capture the relevant activity from the M1 or the lateralized part of the activation represented specifically in the LRP (Leuthold, 2011).

A prerequisite for capturing any electrical brain activity with the EEG is the right orientation of the EEG channel with respect to the dipole source activity: a channel that is positioned directly on top and oriented orthogonally to a dipolar source is unlikely to capture any activity from it (compare Luck, 2014). Due to the LRP being a difference wave (Coles, 1989; Eimer, 1998), it is difficult to test (e.g., by source localization) whether the generator in the M1 motor cortex in this task had this particular location and orientation with respect to the cEEGrid channels. Nevertheless, this hypothesis is in line with the observed results for ERLs recorded at cap electrodes in close proximity to the cEEGrids (i.e., T7/T8): the ERLs recorded over T7/T8 did also not capture the typical dynamics of the LRP (Figures 5C, 6B).

Future studies using specific motor tasks should investigate whether the cEEGrid-EEG can capture symmetrical and asymmetrical parts of motor activation. Here, special attention should be paid to artifact processing in the outer cEEGrid electrodes: visual inspection of Figure 5B indicates that ERLs extracted from cEEGrid-EEG may still be partly contaminated with horizontal eye movements (compare Bleichner and Debener, 2017) thereby possibly obscuring underlying motor activity.

Thus, further validation is needed prior to applying cEEGrid recordings as a tool for monitoring neuronal activity during everyday work or recreational activities. First and foremost, it needs to be carefully examined prior to a study whether the cEEGrid-EEG indeed captures the expected ERPs, cortical asymmetries, or spectral effects of interest. In the context of neuroergonomics, this could refer to both well-known and novel markers of cognitive control (e.g., evoked theta or eye-blink related synchronizations, Wascher et al., 2014) or mental fatigue (e.g., alpha power, Wascher et al., 2015). Moreover, the cEEGrid-EEG’s tolerance against motion artifacts needs to be studied and potentially optimized (Gramann et al., 2011) to be able to monitor naturally-behaving participants in different working environments.

Overall, this study demonstrates the feasibility but also the limitations of cEEGrid recordings for visual tasks. Here, good-quality ERPs and ERLs can be recorded using the cEEGrids. Caution should be executed when recording cEEGrid-EEG during pure motor tasks as response preparation and activation processes may not be adequately reflected in the cEEGrid recordings.

## Author Contributions

EW conceived the design of the study and the approach to the data. MP performed the analysis and wrote the manuscript. Interpretation, revision and final editing of the work were performed by MP, SD and EW.

## Conflict of Interest Statement

The authors declare that the research was conducted in the absence of any commercial or financial relationships that could be construed as a potential conflict of interest.
